# ETV2/ER71 regulates the generation of FLK1^+^ cells from mouse embryonic stem cells through miR-126-MAPK signaling

**DOI:** 10.1186/s13287-019-1466-8

**Published:** 2019-11-19

**Authors:** Ju Young Kim, Dong Hun Lee, Joo Kyung Kim, Hong Seo Choi, Bhakti Dwivedi, Manali Rupji, Jeanne Kowalski, Stefan J. Green, Heesang Song, Won Jong Park, Ji Young Chang, Tae Min Kim, Changwon Park

**Affiliations:** 1 Department of Pediatrics, Emory University School of Medicine, 2015 Uppergate Dr. Atlanta, Atlanta, GA 30322 USA; 2 Children’s Heart Research & Outcomes (HeRO) Center, Children’s Healthcare of Atlanta & Emory University, Atlanta, GA USA; 3 Molecular and Systems Pharmacology Program, Emory University, Atlanta, GA USA; 4 Biochemistry, Cell Biology and Developmental Biology Program, Emory University, Atlanta, GA USA; 5 Winship Cancer Institute, Emory University, Atlanta, GA USA; 6 Department of Biostatistics and Bioinformatics, Emory University, Atlanta, GA USA; 7 Present Address: Department of Oncology, University of Texas at Austin, Austin, TX 78712 USA; 8 Sequencing Core, Research Resources Center, University of Illinois at Chicago, Chicago, IL USA; 9 Department of Biochemistry and Molecular Biology, Chosun University School of Medicine, Gwangju, IL Republic of Korea; 10 Graduate School of International Agricultural Technology and Institute of Green-Bio Science and Technology, Seoul National University, Pyeongchang, Gangwon-do Republic of Korea

**Keywords:** ETV2/ER71, FLK1^+^ cells, miR-126, EGFL7, Embryonic stem cells

## Abstract

Previous studies including ours have demonstrated a critical function of the transcription factor ETV2 (ets variant 2; also known as ER71) in determining the fate of cardiovascular lineage development. However, the underlying mechanisms of ETV2 function remain largely unknown. In this study, we demonstrated the novel function of the miR (micro RNA)-126-MAPK (mitogen-activated protein kinase) pathway in ETV2-mediated FLK1 (fetal liver kinase 1; also known as VEGFR2)^+^ cell generation from the mouse embryonic stem cells (mESCs). By performing a series of experiments including miRNA sequencing and ChIP (chromatin immunoprecipitation)-PCR, we found that miR-126 is directly induced by ETV2. Further, we identified that miR-126 can positively regulate the generation of FLK1^+^ cells by activating the MAPK pathway through targeting SPRED1 (sprouty-related EVH1 domain containing 1). Further, we showed evidence that JUN/FOS activate the enhancer region of FLK1 through AP1 (activator protein 1) binding sequences. Our findings provide insight into the novel molecular mechanisms of ETV2 function in regulating cardiovascular lineage development from mESCs.

## Introduction

The ETS (E26 transformation-specific or E-twenty-six specific sequence) transcription factor family members play key roles in diverse biological events including cell survival, cancer, vascular-angiogenesis and hematopoiesis mainly through the interaction between their conserved ETS DNA binding domain and the consensus sequence (5′-GGAA/T-3′) present in the regulatory elements of their target genes [1, 2]. ETV2 (ets variant 2; also known as ER71), a member of the ETS transcription factor family, has been reported as an indispensable factor in establishing the cardiovascular system [3, 4]. We previously demonstrated that deficiency in *Etv2* led to embryonic lethality due to a complete lack of both vascular and hematopoietic compartments [5, 6]. We also showed that ETV2 can directly bind the promoters/enhancers of endothelial and hematopoietic genes including *Flk1* (fetal liver kinase 1; also known as VEGFR2) [5–7]. Together with the findings that ETV2 can induce de novo generation of FLK1^+^ cells, the multipotent progenitor for blood, endothelial and cardiac lineages from mouse embryonic stem cells (mESCs) [5], these results strongly suggest the critical function of ETV2 for the establishment of the cardiovascular system. Reports from other groups further support the importance of ETV2 in this process [8–11]. Regarding the regulatory mechanisms of ETV2 functions, several studies examining the ETV2 binding proteins have been reported. For example, it was shown that the interaction between ETV2 and FOXC2 (forkhead box protein C2) plays an important role in regulating several key genes of the endothelial and hematopoietic lineages [12, 13]. Also, our recent study revealed the functional significance of the ETV2-OVOL2 (ovo-like zinc finger 2) interaction in generating FLK1^+^ cells and its further differentiation into the hematopoietic and endothelial cells [14]. However, the detailed molecular insight into the ETV2 function remains largely unknown.

To better understand the machinery of ETV2 that regulates the FLK1^+^ cell generation from mESCs, we profiled miRNAs (micro RNAs) that are differentially regulated by ETV2 and found miR-126 as one of the direct downstream players of ETV2. We subsequently investigated the molecular mechanism of the miR-126/MAPK (mitogen-activated protein kinase) pathway in ETV2-mediated FLK1^+^ cell generation.

## Materials and methods

Complete materials and methods are presented in Additional file 1: Supplemental materials and methods.

## Results

### Analysis of ETV2-mediated miRNAs

**Fig. 1:**
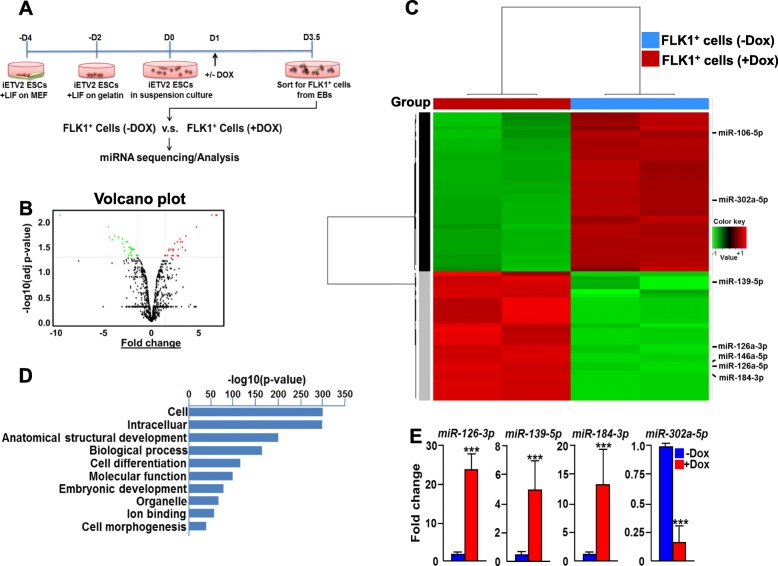
Analysis of ETV2-regualted miRNA expression in FLK1^+^ cells. **a** Schematic diagram of miRNA sequencing experiment. Doxycycline-inducible (iFLAG-ETV2) mESCs were differentiated, treated with ± Doxycycline (Dox) at day 1 and sorted for FLK1^+^ cells at day 3.5. RNAs from the sorted cells were subjected to miRNA sequencing and analysis. **b** Volcano plot showing the log2 fold change between +Dox versus −Dox against the −log10 FDR-adjusted *p* value for each miRNA. miRNAs (FDR ≤ 0.05) with fold change of ≥ 1.5 (in red; upregulated) and ≤ − 1.5 (in green; downregulated) were highlighted and selected. **c** Heatmap of the selected miRNAs in response to overexpression of ETV2. miRNAs upregulated and downregulated in +DOX were indicated with gray and black bars, respectively. **d** Gene Ontology (GO) categories of selected miRNAs by DIANA miRpath analysis. Bars indicate the significance level of miRNA target genes and interactions. **e** Differentiated iFLAG-ETV2 mESCs at day 3.5 were subjected to qRT-PCR analysis. *n* = 3, ***p < 0.001

To gain a novel insight into the molecular mechanisms of ETV2 function in FLK1^+^ cell generation, we performed miRNA profiling analysis. FLK1^+^ cells from doxycycline (Dox)-inducible ETV2 in mESC (herein, iFLAG-ETV2 ESCs) [14] at day 3.5 of differentiation ± Dox were FACS (fluorescence-activated cell sorting)-sorted and subjected to miRNA sequencing (Fig. 1a). The miRNAs with ≥ 1.5 fold change and a false discovery rate (FDR) ≤ 0.05 were considered to be significantly differentially expressed, resulting in a total of 67 miRNAs of interest that were subsequently subjected to unsupervised hierarchical clustering (Fig. 1b, c). GO (gene ontology) term analysis indicated that the ETV2-mediated miRNAs could be involved in diverse biological events with embryo development, cell differentiation and anatomical structure development being top ranked (Fig. 1d). Signaling pathways such as MAPK, RAP1 (ras-associated protein 1) and WNT (wingless-related integration site) were identified as the major regulatory network of the miRNAs, all of which are critical for cardiovascular development (Additional file 2: Tables S1 and S2). Some of the differentially expressed miRNAs were validated by qRT-PCR (Fig. 1e and Additional file 3: Figure S1).

Among the differentially regulated miRNAs, miRNA-126 drew our attention as an important player of ETV2-mediated FLK1^+^ cell generation due to its function in vascular development and hematopoiesis [15–17]. Independently, our previous reports showed that *Egfl7* (egf-like domain multiple 7) [18], the host gene of *miR-126*, is one of the most upregulated genes upon ETV2 overexpression [6, 7]. Accordingly, we sought to determine the functional consequence of miR-126 in regulating ETV2-mediated FLK1^+^ cell generation.

### ETV2 directly activates the expression of miR-126 through *Egfl7* promoter

**Fig. 2:**
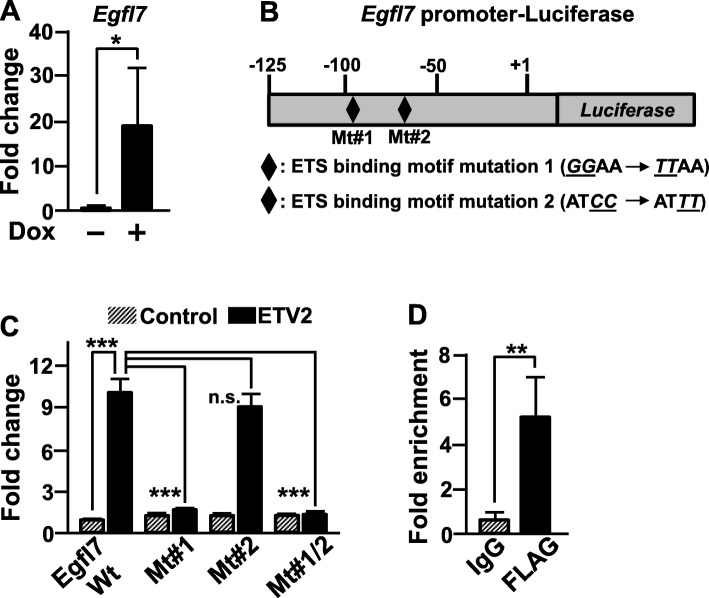
ETV2 upregulates miR126 expression through direct binding on *Egfl7* promoter. **a** Expression analysis. iFLAG-ETV2 mESCs were differentiated for 3.5 days ± Dox treatment and were subjected to gene expression analysis. *n* = 3, *p < 0.05. **b** Schematic diagram of *Egfl7* promoter-luciferase plasmid. Two potential ETS binding elements were marked as diamonds, and their mutant sequences were shown in the bottom. **c** HEK/293T cells were transiently co-transfected with pCMV-*Etv2* and pGL3-luciferase constructs carrying wild type (Wt), or mutants of *Egfl7* promoter. Firefly luciferase activity was normalized by Renilla luciferase activity. *n* = 3. ***p < 0.001. **d** iFLAG-ETV2 mESCs were differentiated in the presence of Dox for 3.5 days and then subjected to ChIP-PCR assay. Rabbit anti-FLAG or IgG antibody was used for the immunoprecipitation. *n* = 3, **p < 0.01

First, we found a significant increment of the expression of both *miR-126* and *Egfl7* in differentiating iFLAG-ETV2 ESCs upon Dox treatment (Figs. 1e and 2a). Next, we examined whether ETV2 can directly activate the promoter of *Egfl7*, thus inducing the expression of miR-126. From the literature search and our own investigation, we found the highly conserved upstream region of the transcription start site in mouse and human EGFL7 that contains two conserved potential ETS binding sites (Fig. 2b). By performing the promoter assay, we revealed that the overexpression of *Etv2* significantly increased the activity of the *Egfl7* promoter. However, the *Egfl7* promoter construct with mutations on one putative ETS site failed to respond to ETV2 (Fig. 2c). The results were further corroborated by chromatin immunoprecipitation (ChIP)-PCR assay, confirming in vivo occupancy of ETV2 in *Egfl7* promoter (Fig. 2d). Taken together, we conclude that the expression of *Egfl7* and thus *miR-126* is directly regulated by ETV2 in differentiating mESCs.

### The miR-126/MAPK pathway plays an important role for ETV2-induced FLK1^+^ cell generation

**Fig. 3:**
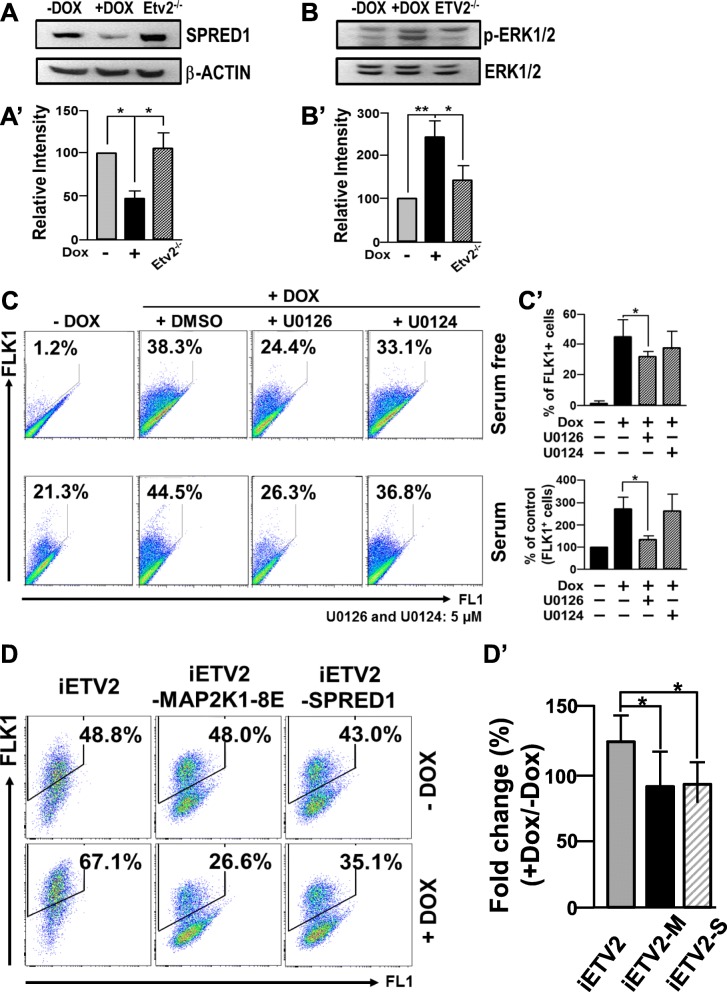
ETV2 increases FLK1^+^ cells through the miR-126/MAPK pathway. **A**, **B** iFLAG-ETV2 mESCs were differentiated with ± Dox for 3.5 days and subjected to western blot analysis for SPRED1 (**A**) and phosphorylated ERK1/2 (**B**). β-ACTIN and ERK1/2 were used as loading controls. (**A’**, **B’**) The relative protein expression of SPRED1 and p-ERK1/2 was normalized against β-ACTIN and total ERK1/2, respectively. Differentiated *Etv2*^*−/−*^ mESCs were also included for the analysis. *n* = 3, *p < 0.05, **p < 0.01. (**C**) iFLAG-ETV2 mESCs were differentiated for 4 days in serum-free (upper) or for 3.5 days in serum (lower) conditions in the presence or absence of Dox. The resulting cells were analyzed for FLK1 expression by flow cytometry. U0126 (5 μM) or U0124 (5 μM) was treated during the differentiation. **C’** Quantification. *n* = 3, *p < 0.05. **D** Differentiated iFLAG-ETV2, iFLAG-ETV2-MAP2K1-8E (iFLAG-ETV2-M) or iFLAG-ETV2-SPRED1 (iFLAG-ETV2-S) mESC ± Dox were analyzed for FLK1 expression by flow cytometry. **D’** Quantification. *n* = 3, *p < 0.05

MiR-126 (especially, miR-126-3p) can regulate the MAPK pathway through the suppression of the SPRED1 expression, a negative regulator of the MAPK pathway [15, 19]. Together with the report that treatment of bFGF (basic fibroblast growth factor), an agonist of FGF receptor-mediated signaling including MAPK [20], increases the generation of the FLK1^+^ cells from mESCs [21], we hypothesized that ETV2 induces FLK1^+^ cells partly through the miR-126-MAPK pathway by suppressing the expression of SPRED1. As shown in Fig. 3a and b, while SPRED1 (sprouty related EVH1 domain containing 1) was significantly reduced in response to ETV2, augmented phospho-ERK1/2 (extracellular signal-regulated kinases 1/2) level was evident upon the ETV2 overexpression in differentiating mESCs. Interestingly, *Etv2*^*−/−*^ ESCs showed an increased level of SPRED1 expression with concomitant decrease of phosphorylated ERK1/2. Further, we found that ETV2-induced FLK1^+^ cell generation was significantly reduced in the presence of U0126, a MAPK inhibitor, compared to the control (Fig. 3c). To corroborate the findings, we transfected Dox-inducible MAP2K1-8E, a catalytically inactive form of MAP2K1 [22] into iFLAG-ETV2 mESCs in which both ETV2 and MAP2K1-8E are co-overexpressed upon Dox treatment. In agreement with the inhibitor treatment (Fig. 3c), the co-expression of MAPK2K1-8E and ETV2 led to a decreased generation of FLK1^+^ cells, compared to the group in which ETV2 only is overexpressed (Fig. 3d). Additionally, we went on to show that overexpression of SPRED1 was able to inhibit generation of FLK1^+^ cells upon the overexpression of ETV2 (Fig. 3d). These results clearly suggest that the SPRED1/MAPK pathway plays an important role for ETV2-induced FLK1^+^ cell generation from mESCs.

### Direct activation of FLK1 gene expression by the MAPK pathway

**Fig. 4:**
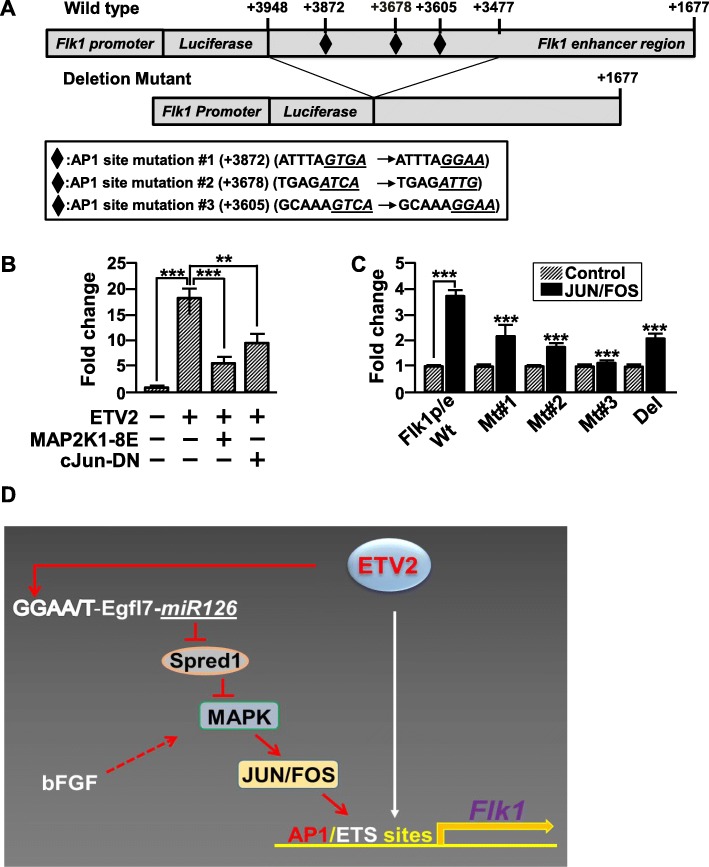
ETV2 activates FLK1 expression through AP-1 binding sites in Flk1 enhancer region. **a** Schematic diagram of *Flk1*-promoter-enhancer (p/e)-luciferase plasmid. Three potential AP-1 binding elements were marked as diamonds. **b**, **c** Luciferase-based promoter assay. **b** pGL3-*Flk1* p/e was transiently co-transfected with pCMV-*Etv2,* pMCL-*MAP2K1-8E*, pCMV-*c-Jun* ND (dominant negative form of c-Jun) into HEK/293T cells. *n* = 3, **p < 0.01, ***p < 0.001. **c** pGL3-luciferase construct carrying wild type (Wt), putative AP-1 binding site mutants (Mt #1, Mt #2, Mt #3) or enhancer deletion mutant (del) of *Flk1* p/e was co-transfected with pCMV-*c-Jun and c-Fos* into HEK/293T cells. Firefly luciferase activity was normalized by Renilla luciferase activity. *n* = 3, ***p < 0.001. **d** A working model for ETV2-miR126/MAPK in regulating FLK1 expression. In addition to the direct binding of ETV2 on the ETS binding sites in FLK1 gene, ETV2 activates miR-126, which can target SPRED1, thereby activating the MAPK pathway. JUN/FOS, a downstream signaling complex of the MAPK pathway, subsequently activates gene transcription of FLK1 via the AP1 binding sites

To get a detailed insight into how the MAPK pathway activated by ETV2 regulates the generation of FLK1^+^ cells, we examined the regulatory elements such as promoter and enhancer of *Flk1* gene [23] and found several potential activator protein 1 (AP1) binding sequences in *Flk1* enhancer which is critical for controlling the endogenous expression of *Flk1* (Fig. 4a). AP1 (activator protein 1), a heterodimeric transcriptional complex consisting of JUN and FOS proteins, conveys the important functions for multiple biological processes such as differentiation, proliferation and apoptosis [24]. Given the findings that AP1 complex acts as a downstream target of the MAPK pathway [25], these suggest an important mechanistic link between ETV2 and FLK1 gene expression via the MAPK-AP1 pathway. Therefore, we first performed a luciferase-based promoter assay and revealed that the activity of the *Flk1-*promoter/enhancer (*p/e*) was increased in response to ETV2 or c-JUN/FOS, a downstream of the MAPK pathway (Fig. 4b, c). In contrast, both MKAP2K1-8E and the JUN dominant negative mutant, c-JUN DN [26], inhibited ETV2 function in activating *Flk1-p/e* (Fig. 4b). Further, we showed that mutations on the putative AP-1 binding site in the *Flk1-p/e* led to a significantly reduced luciferase activity induced by c-JUN/FOS (Fig. 4c). Collectively, these results suggest that the MAPK pathway can activate the expression of *Flk1* gene partly through c-JUN/FOS.

## Discussion

In our study, several layers of novel findings on ETV2 function were made. First, we reported genome-wide miRNA profiles in FLK1^+^ cells generated in response to ETV2, providing a new research resource. Second, we demonstrated the functional significance of the miR-126/MAPK pathway in ETV2-mediated FLK1^+^ cell generation. Third, we also showed a direct activation of *Flk1* enhancer by the JUN/FOS complex. Overall, our results reveal that the miR-126/MAPK pathway constitutes a novel mechanism responsible for ETV2-mediated *Flk1* expression (Fig. 4d).

Our miRNA profiling results suggest that ETV2 regulates development of FLK1^+^ cells, hematopoietic and endothelial cell lineages as well as cardiomyocytes through miRNAs. For example, miR-10b, one of the downregulated miRNAs by ETV2, is a critical regulator for vessel development through targeting FLT-1 (fms-related tyrosine kinase 1), which can inhibit VEGF (vascular endothelial growth factor)-FLK1 signaling [27]. In contrast, miR-146a was upregulated in response to ETV2 expression. Interestingly, miR-146 can target CXCR4 (C-X-C chemokine receptor type 4), a receptor of SDF1 (stromal cell-derived factor 1), whose signaling is important during heart development and promotes cardiogenesis from pluripotent stem cells [28–31]. Another downregulated miRNA by ETV2, miR-106, has been reported as a critical player for cardiac development as evidenced by in utero lethality displaying severe cardiac defects in compound knockout of mouse miR-106~25 and miR-17~92 cluster [32]. Thus, determining biological consequences of the links between ETV2 and ETV2-dependent miRNAs for cardiovascular lineage development would be an interesting topic for further studies.

Studies have shown that miR-126 plays important functions in cardiovascular development and angiogenesis [15–17]. In this study, we provided evidence of the unknown function of miR-126 in generating the first emerging FLK1^+^ cells. Our results showed that ETV2 via a direct binding on the *Egfl7* promoter can induce the expression of miR-126. In the subsequent studies, inhibition of the miR-126/MAPK pathway leads to impairment of ETV2-mediated FLK1^+^ cell generation. Thus, our results suggest a novel post-transcriptional regulatory mechanism of ETV2 in generating FLK1^+^ cell. Although EGFL7 is co-transcribed with miR-126 upon ETV2 expression, we rule out the potential contribution of EGFL7 to inducing FLK1^+^ cell generation due to the dispensable function of EGFL7 in early stages of embryogenesis and embryonic vasculature formation as demonstrated by knockout studies in mice and knockdown experiments in differentiating ESCs [18, 33]. Taken together, it is evident that miR-126 acting downstream of ETV2 plays important roles in the establishment of the cardiovascular system.

The molecular insights into *Flk1* gene expression (i.e., determining its upstream regulators) has not been an active research area, despite its critical function in hematopoietic and vascular system. Regarding this, we have demonstrated that ETV2 directly binds ETS binding elements present on both promoter and enhancer of *Flk1* and activates its expression [5]. The subsequent ChIP sequencing analysis demonstrated that a wide range of genes critical for endothelial cell and blood cells are direct downstream targets of ETV2 [7]. In this current study, we revealed an important function of MAPK activity in regulating *Flk1* gene expression. Overexpression of ETV2 activates the MAPK activity, while inhibition of the MAPK pathway decreases ETV2-mediated FLK1^+^ cell generation. Further, we showed that the MAPK pathway is able to directly upregulate the expression of Flk1 via a direct binding of JUN/FOS on *Flk1* enhancer. Thus, we envision that ETV2 induces *Flk1* gene expression in a bimodal manner, one by activating the miR-126/MAPK pathway and the other by its direct binding on *Flk1* gene regulatory elements. In mouse embryos, activated ERK1/2 is detected in the blood islands, the first sites of blood and endothelial cell development and later in the dorsal aorta and intersomitic vessels [34]. Further, *Erk2*-deficient mouse embryos are incompatible with proper mesoderm formation [35, 36]. Sprouty/Spred proteins, the negative effectors for Ras/MAPK pathway [19], have inhibitory functions in generating mesoderm and blood as well as vessel development [37–40], further suggesting important functions of the MAPK pathway in emergence of mesoderm with hematopoietic and endothelial potential. Considering that the enhanced protein stability of ETV2 by OVOL2 is thought to be one mechanism for ETV2-mediated *Flk1* gene transcription [14], it would be interesting to see if the interaction between ETV2 and OVOL2 can positively regulate the miR-126/MAPK pathway in this process. In addition, the findings that ETV2 interacts with OVOL2 [14] or FOXC2 [12] and that ETV2 and JUN/FOS acts on *Flk1* promoter and enhancer suggest a hypothesis that ETV2 can form a transcriptional complex with OVOL2, FOXC2, and/or AP1 to regulate the expression of *Flk1*, which could provide an in-depth and novel insight into molecular mechanisms behind the ETV2-FLK1 axis.

In conclusion, we reported miRNA profiles regulated by ETV2 in generating FLK1^+^ cells from mESCs. Further, we showed that ETV2 can regulate the expression of Flk1 through the miR126/MAPK pathway. These findings could provide a novel insight into the mechanisms of how ETV2 regulates the development of the cardiovascular system. Studying the functions of other miRNAs identified in this research would be important for further deciphering the ETV2-miRNA regulatory mechanisms in FLK1^+^ cell and cardiovascular lineage generation. Since embryonic events can often play a critical role in adults, examination of our findings in pathophysiological angiogenesis would be warranted.

## Supplementary information

**Additional file 1.** Supplemental materials and methods

**Additional file 2: Table S1.** Gene Ontology (GO) categories for genes targeted by significant differentially expressed miRNA’s. **Table S2**. KEGG categories for genes targeted by significant differentially expressed miRNA’s. **Table S3**. Primer sequences.

**Additional file 3: Figure S1.** Analysis on miR-126-5p in response to ETV2. Differentiated iFLAG-ETV2 mESCs at day 3.5 were subjected to qRT-PCR analysis. *n*=3, **p<0.01.

## Data Availability

The datasets used and/or analyzed during the current study are available from the corresponding author on reasonable request.

## Abbreviations

AP1: Activator protein 1
ChIP: Chromatin immunoprecipitation
c-JUN DN: JUN dominant negative mutant
CXCR4: C-X-C chemokine receptor type 4
Dox: Doxycycline
EGFL7: Egf-like domain multiple 7
ERK1/2: Extracellular signal-regulated kinases 1/2
ETS: E26 transformation-specific
ETV2: Ets variant 2
FACS: Fluorescence-activated cell sorting
FDR: False discovery rate
FLK1: Fetal liver kinase 1
Flk1-p/e: Flk1-promoter/enhancer
FLT-1: Fms-related tyrosine kinase 1
FOXC2: Forkhead box protein C2
GO: Gene ontology
iFLAG-ETV2: Doxycycline-inducible FLAG-ETV2
MAPK: Mitogen-activated protein kinase
mESCs: Mouse embryonic stem cells
OVOL2: Ovo-like zinc finger 2
RAP1: Ras-associated protein 1
SDF1: Stromal cell-derived factor 1
SPRED1: Sprouty-related EVH1 domain containing 1
VEGF: Vascular endothelial growth factor
WNT: Wingless-related integration site
