# Supplementary material for: ETV2/ER71 regulates the generation of FLK1+ cells from mouse embryonic stem cells through miR-126-MAPK signaling
Source: Stem Cell Res Ther. 2019 Nov 19;10:328. doi: 10.1186/s13287-019-1466-8 (PMC6862833; doi:10.1186/s13287-019-1466-8)
Supplement: Supplementary file 1 — Additional file 1. Supplemental materials and methods [file 13287_2019_1466_MOESM1_ESM.docx]

**Supplemental materials and methods**

**mESC culture and differentiation**

Maintenance and differentiation of Dox-inducible FLAG-ETV2 (iFLAG-ETV2) [1] and *Etv2*^-/-^ mESCs [2] were performed as described previously [1, 3-5]. SPRED1 cDNA (Open Biosystems, Lafayette, CO) and MAP2K1-8E cDNA (Addgene plasmid # 40811) [6] were cloned into pFUW-tet-O-MCS (addgene plasmid #84008) [7], respectively. The resulting plasmids were transfected into iFLAG-ETV2 mESC, followed by ESC differentiation assay. During the differentiation, Dox (1 µg/ml), U0126 (5 µM, Tocris, Bristol, United Kingdom) and U0124 (5 µM, Tocris, Bristol, United Kingdom) were treated as described previously [5].

**Cell sorting and flow cytometry analysis**

Dissociated cells with StemPro Accutase (Thermo Fisher Scientific, Grand Island, NY) were stained with PE-conjugated anti-mouse FLK1 monoclonal antibody (BioLegend, San Diego, CA) in FACS buffer (4% FCS in PBS) [1, 3-5], followed by cell sorting with FACS ARIA II (Becton-Dickinson, Franklin Lakes, NJ). For flow cytometry analysis, samples were prepared as in the same way as the cell sorting, and subjected to flow cytometry analysis with a BD LSRII (Beckton-Dickinson, Franklin Lakes, NJ). Data were analyzed with FlowJo (Vers.10).

**Construction of small RNA libraries**

Samples were prepared in the same manner as for cell sorting. RNA was isolated from the cells by using Trizol (Invitrogen, Carlsbad, CA). Construction of libraries and sequencing on the Illumina HiSeq2500 were performed at the W. M. Keck Center for Comparative and Functional Genomics at the University of Illinois at Urbana-Champaign. Small RNA libraries were constructed from the RNA isolated from sorted FLK1^+^ cells using the TruSeq small RNA Sample Preparation Kit (Illumina, San Diego, CA). The individually-barcoded libraries were mixed into a pool, which was sized and selected on a Novex 10% TBE gel (LifeTechnologies, Carlsbad, CA) to enrich for small RNAs 17 nt to 30 nt in length. The final libraries were quantitated by Qubit (Life Technologies, Carlsbad, CA) and the average size was determined on an Agilent Bioanalyzer High Sensitivity DNA chip (Agilent Technologies, Santa Clara, CA) and diluted to 10 nM. The pooled libraries were further quantitated by qPCR on an ABI 7500 (Life Technologies, Carlsbad, CA).

**RNA Sequencing**

The pooled libraries were loaded onto one lane (2012) and two lanes (2013) of an 8-lane flowcell for cluster formation and sequenced on an Illumina HiSeq 2000 (2012) and HiSeq 2500 (2013). One of the lanes was loaded with a PhiX Control library that provides a balanced genome for calculation of matrix, phasing, and pre-phasing. The libraries were sequenced from one end of the molecules to a total read length of 50 nt. The raw .bcl files were converted into demultiplexed fastq files with Casava 1.8.2 (Illumina, [San Diego, CA](https://www.google.com/search?client=firefox-b-1&q=San+Diego&stick=H4sIAAAAAAAAAOPgE-LSz9U3MDIvMUxPUeIAsc0Ny4q0tLKTrfTzi9IT8zKrEksy8_NQOFYZqYkphaWJRSWpRcWLWDmDE_MUXDJT0_MBY_F3uVAAAAA&sa=X&ved=2ahUKEwiiuouJnMjgAhUmUt8KHaRZB6QQmxMoATAvegQICBAL&biw=1920&bih=912)).

**miRNA sequencing data analysis**

The quality of the single-ended miRNA sequencing data was assessed using FastQC (<http://www.bioinformatics.babraham.ac.uk/projects/fastqc>). The 3’-adaptor and low-quality sequences were trimmed using cutadapt (v 1.4.2) [8] with the command options: cutadapt -a TGGAATTCTCGGGTGCCAAGGAACTCCAGTCAC -O 5 -m 16 -q 30 --discard-untrimmed <raw_reads_fastq_file> <trimmed_reads_fasta_file>. The length distribution of resulting reads was centered around 22 nt and 32 nt. The trimmed reads were mapped to the mouse reference genome (GRCm38/mm10; <https://genome.ucsc.edu/index.html>) and analyzed using miRDeep2 package (v2.0.0.8) [9]. The reads were preprocessed using mapper module of miRDeep2 with the command options: perl mapper.pl <trimmed_collapsed_reads_fasta_file> -e -h -j –k TGGAATTCTCGGGTGCCAAGGAACTCCAGTCAC -l 17 -m -p <mm10_cluster> -s <reads_fasta_file> -t <trimmed_collapsed_vs_genome.arf> –v. The miRNA expression levels of known mature miRNAs were measured using the quantifier module with command options: perl quantifier.pl -p <mm10_hairpin.fa> -m <mm10_mature.fa> -r <trimmed_collapsed_reads_fasta_file> -t Mouse -y <output_file>. The novel miRNAs were also predicted using the miRDeep2 module with command options: perl miRDeep2.pl <trimmed_collapsed_reads_fasta_file> <mm10_chromFa_1_22.fa> <trimmed_collapsed_vs_genome.arf> <mm10_mature.fa> none <mm10_hairpin.fa> -t Mouse. Novel miRNAs with significant randfold p-value were kept for further analysis. All published mature miRNA precursors and hairpins for the mouse genome were downloaded from miRBase release 21 (<http://www.mirbase.org/>). The raw expression counts from both known and novel miRNAs for all four samples were normalized using DESeq package [10] and log2 transformed. A total of 2240 known and 119 novel miRNAs were detected.

**Differential expression analysis of miRNAs**

Differentially expressed miRNAs were identified using the Moderated t-test available in ‘limma’ R package [11]. An FDR adjusted p-value was calculated and applied. The miRNAs with ≥ 1.5 fold change and an FDR ≤ 0.05 were considered to be significantly differentially expressed, resulting in a total of 67 known miRNAs (30 up-regulated and 37 down-regulated). Unsupervised hierarchical clustering was performed with 1-pearson correlation distance and average linkage clustering method as implemented in NOJAH (<http://bbisr.shinyapps.winship.emory.edu/NOJAH/)>.

**Functional annotation analysis of significant known miRNAs**

Predicted miRNA target genes and interactions were identified using the DIANA-miRPath (v3.0) [12] by the DIANA-microT-CDS algorithm. KEGG and GO analyses were conducted on the predicted targets of known 22 up-regulated and known 33 down-regulated differentially expressed miRNAs.

**Real time quantitative reverse transcription PCR**

One µg of total RNA prepared using Trizol (Thermo Fisher Scientific, Grand Island, NY) was used for cDNA synthesis, followed by real time PCR as described previously [1]. For miRNAs, cDNAs were generated using miScript PCR starter kit (Qiagen, Venlo, Netherlands) and the expression of miRNAs was determined according to the manufacture’s instruction (Qiagen, Venlo, Netherlands). Each experiment with duplicates was performed at least three times. Primer sequences are in additional file 3: Table S3.

**Luciferase assay**

HEK/293T cells (4 x 10^4^ cells/well/12-well plate) were co-transfected with 40 ng of p-RL-null (Promega, Madison, WI), 200 ng of each Firefly reporter construct in combinations with 1.2 µg of expression plasmids of either ETV2, MAP2K1-8E (Addgene plasmid # 40811) [6] , c-JUN, c-FOS or c-JUN DN (dominant negative form of c-Jun, Addgene plasmid # 40350) [13] using EndoFectin™ Max (GeneCopoeia, Rockville, MD). Cells were collected after 48 hours of transfection, and luciferase activity was measured with Dual-Luciferase reporter assay system according to the manufacturer’s instruction (Promega, Madison, WI) [1, 4]. Firefly luciferase activity was normalized to Renilla luciferase activity to control transfection efficiency. Each experiment with duplicates was performed at least three times. The mutant reporter plasmids (Egfl7 and Flk1 p/e) were generated with site-directed mutagenesis kit according to the manufacture’s instruction (Agilent Technologies, Santa Clara, CA). The primer sequences for constructing the reporter plasmids are listed in additional file 3: Table S3.

**Chromatin immunoprecipitation assay**

The procedure was previously described [4]. Briefly, iFLAG-ETV2 at day 3.5 of mESCs differentiation in the presence of Dox were cross-linked with 1% formaldehyde and lysed with cell membrane lysis buffer [5 mM PIPES, 85 mM KCl, 0.5% NP-40, Protease inhibitor cocktail (Sigma-Aldrich, St. Louis, MO)]. The resulting nuclear fraction was further lysed with nuclear membrane lysis buffer (10 mM EDTA, 1% SDS in Tris-HCl) and subjected to sonication with Bioruptor (Diagenode, Denville, NJ). The solution containing sonicated chromatin was precleared with protein A/G PLUS agarose beads (Santa Cruz Biotechnology, Inc. Dallas, TX) and the supernatant was subsequently incubated with 5 µg of rabbit anti-FLAG antibody (Sigma-Aldrich, St. Louis, MO) or normal rabbit IgG (Santa Cruz Biotechnology, Inc. Dallas, TX). Immunoprecipitated DNA fragments were isolated and subjected to quantitative PCR to measure protein binding on *Egfl7* promoter. Primer sequences are listed in additional file 3: Table S3.

**Western blot analysis**

Briefly, cells were lysed with the RIPA buffer containing phosphatase inhibitor cocktail, protease inhibitors (Sigma-Aldrich, St. Louis, MO) and 0.1 M PMSF. The lysates were then subjected to SDS-PAGE, followed by a Western analysis with rabbit anti-phospo-ERK1/2 (1:1000) (Cell Signaling Technology, Danvers, MA), rabbit anti-ERK1/2 (1:1000) (Cell Signaling Technology, Danvers, MA), rabbit anti-SPRED1 (1:1000) (Millipore Sigma, Burlington, MA), or mouse anti-β-ACTIN antibody (Sigma-Aldrich, St. Louis, MO).

**Statistical analysis**

All data were analyzed by GraphPad Prism 7 software for statistical analysis and generating graphs/plots. Data were analyzed by one-way ANOVA with post hoc Dunnett’s, Tukey’s or Bonferroni’s multiple-comparison test. Statistical differences between two groups were determined by two-tailed Student’s *t* test. *P* < 0.05 was considered statistically significant.

**Supplemental references**

1. Kim JY, Lee RH, Kim TM, Kim DW, Jeon YJ, Huh SH, Oh SY, Kyba M, Kataoka H, Choi K, Ornitz DM, Chae JI, Park C. OVOL2 is a critical regulator of ER71/ETV2 in generating FLK1+, hematopoietic, and endothelial cells from embryonic stem cells. Blood. 2014;124(19):2948-52.

2. Liu F, Kang I, Park C, Chang LW, Wang W, Lee D, Lim DS, Vittet D, Nerbonne JM, Choi K. ER71 specifies Flk-1+ hemangiogenic mesoderm by inhibiting cardiac mesoderm and Wnt signaling. Blood. 2012;119(14):3295-305.

3. Ma YD, Lugus JJ, Park C, Choi K. Differentiation of mouse embryonic stem cells into blood. Curr Protoc Stem Cell Biol. 2008;Chapter 1:Unit 1F 4.

4. Lee D, Park C, Lee H, Lugus JJ, Kim SH, Arentson E, Chung YS, Gomez G, Kyba M, Lin S, Janknecht R, Lim DS, Choi K. ER71 acts downstream of BMP, Notch, and Wnt signaling in blood and vessel progenitor specification. Cell Stem Cell. 2008;2(5):497-507.

5. Park C, Afrikanova I, Chung YS, Zhang WJ, Arentson E, Fong Gh G, Rosendahl A, Choi K. A hierarchical order of factors in the generation of FLK1- and SCL-expressing hematopoietic and endothelial progenitors from embryonic stem cells. Development. 2004;131(11):2749-62.

6. Mansour SJ, Matten WT, Hermann AS, Candia JM, Rong S, Fukasawa K, Vande Woude GF, Ahn NG. Transformation of mammalian cells by constitutively active MAP kinase kinase. Science. 1994;265(5174):966-70.

7. Panciera T, Azzolin L, Fujimura A, Di Biagio D, Frasson C, Bresolin S, Soligo S, Basso G, Bicciato S, Rosato A, Cordenonsi M, Piccolo S. Induction of Expandable Tissue-Specific Stem/Progenitor Cells through Transient Expression of YAP/TAZ. Cell Stem Cell. 2016;19(6):725-37.

8. Martin M. Cutadapt removes adapter sequences from high-throughput sequencing reads. 2011. 2011;17(1):3.

9. Friedlander MR, Mackowiak SD, Li N, Chen W, Rajewsky N. miRDeep2 accurately identifies known and hundreds of novel microRNA genes in seven animal clades. Nucleic Acids Res. 2012;40(1):37-52.

10. Love MI, Huber W, Anders S. Moderated estimation of fold change and dispersion for RNA-seq data with DESeq2. Genome Biol. 2014;15(12):550.

11. Ritchie ME, Phipson B, Wu D, Hu Y, Law CW, Shi W, Smyth GK. limma powers differential expression analyses for RNA-sequencing and microarray studies. Nucleic Acids Res. 2015;43(7):e47.

12. Vlachos IS, Zagganas K, Paraskevopoulou MD, Georgakilas G, Karagkouni D, Vergoulis T, Dalamagas T, Hatzigeorgiou AG. DIANA-miRPath v3.0: deciphering microRNA function with experimental support. Nucleic Acids Res. 2015;43(W1):W460-6.

13. Wang ZY, Sato H, Kusam S, Sehra S, Toney LM, Dent AL. Regulation of IL-10 gene expression in Th2 cells by Jun proteins. J Immunol. 2005;174(4):2098-105.
