# Supplementary material for: ETV2/ER71 regulates the generation of FLK1+ cells from mouse embryonic stem cells through miR-126-MAPK signaling
Source: Stem Cell Res Ther. 2019 Nov 19;10:328. doi: 10.1186/s13287-019-1466-8 (PMC6862833; doi:10.1186/s13287-019-1466-8)
Supplement: Supplementary file 2 — Additional file 2: Table S1. Gene Ontology (GO) categories for genes targeted by significant differentially expressed miRNA’s. Table S2. KEGG categories for genes targeted by significant differentially expressed miRNA’s. Table S3. Primer sequences. [file 13287_2019_1466_MOESM2_ESM.docx]

**Table S1.** Gene Ontology (GO) categories for genes targeted by significant differentially expressed miRNA’s

| **GO Category** | **p-value** | **#genes** | **#miRNAs** | **"-log10(p-value)"** |
| --- | --- | --- | --- | --- |
| cell | 0.0000 | 3622 | 49 | 300 |
| intracellular | 0.0000 | 3127 | 49 | 300 |
| anatomical structure development | 0.00 | 1227 | 49 | 200.2325422 |
| biological_process | 0.00 | 4980 | 49 | 164.0368007 |
| cell differentiation | 0.00 | 930 | 48 | 114.5668795 |
| molecular_function | 0.00 | 4899 | 49 | 98.20152183 |
| embryo development | 0.00 | 376 | 46 | 77.33462401 |
| organelle | 0.00 | 2617 | 49 | 65.7082608 |
| ion binding | 0.00 | 1705 | 49 | 56.28982137 |
| cell morphogenesis | 0.00 | 263 | 48 | 38.02924933 |
| cellular_component | 0.00 | 4766 | 49 | 36.51354602 |
| anatomical structure formation involved in morphogenesis | 0.00 | 273 | 48 | 32.31528644 |
| cellular protein modification process | 0.00 | 692 | 49 | 30.31555809 |
| chromosome organization | 0.00 | 208 | 46 | 29.5125585 |
| nucleic acid binding transcription factor activity | 0.00 | 337 | 47 | 25.06050677 |
| cellular nitrogen compound metabolic process | 0.00 | 1178 | 48 | 20.92612194 |
| biosynthetic process | 0.00 | 1018 | 48 | 15.90441128 |
| developmental maturation | 0.00 | 69 | 39 | 14.79550963 |
| cell motility | 0.00 | 204 | 45 | 14.79550963 |
| growth | 0.00 | 144 | 45 | 10.07327537 |
| homeostatic process | 0.00 | 251 | 48 | 8.786299766 |
| cell division | 0.00 | 156 | 46 | 7.987821828 |
| nuclear chromosome | 0.00 | 79 | 42 | 7.502357625 |
| cytoskeleton organization | 0.00 | 209 | 46 | 7.018996617 |
| protein complex | 0.00 | 942 | 49 | 3.693859647 |
| cytoplasmic membrane-bounded vesicle | 0.00 | 145 | 45 | 3.531673952 |
| cytoskeleton | 0.00 | 399 | 46 | 3.365295015 |
| protein binding transcription factor activity | 0.00 | 121 | 45 | 2.906553185 |
| chromosome | 0.00 | 178 | 45 | 2.886197577 |
| cell cycle | 0.00 | 267 | 47 | 2.853689782 |
| circulatory system process | 0.00 | 49 | 40 | 2.765379044 |
| in utero embryonic development | 0.01 | 109 | 43 | 1.895598009 |
| cell death | 0.02 | 222 | 46 | 1.740161076 |
| cell-cell signaling | 0.02 | 163 | 47 | 1.6949872 |
| cellular component assembly | 0.02 | 302 | 48 | 1.673072596 |
| cytoskeletal protein binding | 0.04 | 186 | 45 | 1.44413659 |
| vasculogenesis involved in coronary vascular morphogenesis | 0.04 | 7 | 15 | 1.390190127 |

**Table S2.** KEGG categories for genes targeted by significant differentially expressed miRNA’s

| **KEGG pathway** | **p-value** | **#genes** | **#miRNAs** | **"-log10(p-value)"** |
| --- | --- | --- | --- | --- |
| Proteoglycans in cancer | 6.50E-12 | 88 | 42 | 11.18688633 |
| Prion diseases | 3.86E-11 | 10 | 14 | 10.41385636 |
| Axon guidance | 1.03E-09 | 61 | 35 | 8.9868105 |
| Endocytosis | 9.60E-07 | 86 | 39 | 6.017761907 |
| Rap1 signaling pathway | 1.45E-06 | 86 | 42 | 5.839167396 |
| Renal cell carcinoma | 1.00E-05 | 34 | 39 | 4.998721479 |
| Phosphatidylinositol signaling system | 2.18E-05 | 37 | 31 | 4.661629524 |
| cAMP signaling pathway | 3.91E-05 | 79 | 44 | 4.408276715 |
| Glioma | 4.24E-05 | 28 | 34 | 4.373060948 |
| MAPK signaling pathway | 5.42E-05 | 95 | 44 | 4.265664646 |
| Amphetamine addiction | 5.57E-05 | 32 | 31 | 4.25388316 |
| Long-term potentiation | 6.95E-05 | 34 | 32 | 4.158242385 |
| mTOR signaling pathway | 0.000114392 | 32 | 34 | 3.941604808 |
| Regulation of actin cytoskeleton | 0.000114392 | 80 | 42 | 3.941604808 |
| Pathways in cancer | 0.000127938 | 137 | 45 | 3.893001494 |
| Long-term depression | 0.000132455 | 24 | 26 | 3.877930067 |
| Hippo signaling pathway | 0.000132455 | 54 | 41 | 3.877930067 |
| Wnt signaling pathway | 0.000135392 | 56 | 35 | 3.868407797 |
| Choline metabolism in cancer | 0.00015993 | 44 | 38 | 3.796069707 |
| Signaling pathways regulating stem cells pluripotency | 0.000159991 | 54 | 31 | 3.795905373 |
| Neurotrophin signaling pathway | 0.000224838 | 51 | 39 | 3.648129916 |
| Ras signaling pathway | 0.000224838 | 83 | 41 | 3.648129916 |
| Pancreatic cancer | 0.00057873 | 31 | 32 | 3.237523636 |
| Adherens junction | 0.000658241 | 32 | 34 | 3.181615285 |
| TGF-beta signaling pathway | 0.000691735 | 33 | 35 | 3.160060191 |
| Focal adhesion | 0.000740932 | 76 | 42 | 3.130221605 |
| FoxO signaling pathway | 0.000817288 | 55 | 39 | 3.087625065 |
| Oxytocin signaling pathway | 0.000894906 | 59 | 36 | 3.048222471 |
| Fc gamma R-mediated phagocytosis | 0.001136757 | 37 | 33 | 2.944332204 |
| Dorso-ventral axis formation | 0.001498489 | 15 | 28 | 2.824346524 |
| Melanogenesis | 0.001558336 | 39 | 27 | 2.807338972 |
| cGMP-PKG signaling pathway | 0.001558336 | 63 | 40 | 2.807338972 |
| PI3K-Akt signaling pathway | 0.001558336 | 115 | 42 | 2.807338972 |
| Thyroid hormone signaling pathway | 0.001829502 | 41 | 31 | 2.737667038 |
| ErbB signaling pathway | 0.001968611 | 36 | 38 | 2.705840083 |
| Estrogen signaling pathway | 0.003302 | 34 | 33 | 2.481222943 |
| Thyroid hormone synthesis | 0.003714302 | 23 | 24 | 2.430122735 |
| T cell receptor signaling pathway | 0.004493647 | 40 | 37 | 2.347401003 |
| Inositol phosphate metabolism | 0.004965342 | 26 | 27 | 2.304050874 |
| Colorectal cancer | 0.006487366 | 26 | 28 | 2.187931572 |
| GABAergic synapse | 0.006997878 | 28 | 23 | 2.155033632 |
| Prostate cancer | 0.008388731 | 34 | 36 | 2.076303716 |
| Platelet activation | 0.012759285 | 46 | 36 | 1.894173661 |
| Fc epsilon RI signaling pathway | 0.017380323 | 28 | 33 | 1.759942156 |
| Glutamatergic synapse | 0.018594838 | 40 | 28 | 1.730607604 |
| Dopaminergic synapse | 0.020273867 | 49 | 33 | 1.693063407 |
| GnRH signaling pathway | 0.020273867 | 33 | 35 | 1.693063407 |
| Lysine biosynthesis | 0.020769999 | 2 | 2 | 1.682563526 |
| Osteoclast differentiation | 0.022897334 | 43 | 33 | 1.640215079 |
| Hepatitis B | 0.028927905 | 43 | 34 | 1.538683024 |
| Transcriptional misregulation in cancer | 0.028927905 | 54 | 39 | 1.538683024 |
| B cell receptor signaling pathway | 0.029122773 | 27 | 32 | 1.535767276 |
| Sphingolipid signaling pathway | 0.032896149 | 42 | 32 | 1.482854941 |
| Vascular smooth muscle contraction | 0.037441826 | 41 | 33 | 1.426642982 |
| Calcium signaling pathway | 0.046786024 | 58 | 36 | 1.329883857 |

| **qPCR** |  | **Sequences (5’-3’)** | **Comments** |
| --- | --- | --- | --- |
| *Egfl7*  *qRT-PCR* | Forward | CGCTGTGTCAATACTGTGGGA | Primer Bank  ID :257096015c3 |
|  | Reverse | GTTCTAGCACATCAACCCGAG |  |
| *Egfl7 ChIP-PCR* | Forward | CCCTTGGCCTCCTGTTTGTT |  |
|  | Reverse | AGCCAACCATGCAATGCAAC |  |
| **Cloning** |  | **Sequences (5’-3’)** |  |
| *Egfl7 promoter* | Forward | CATGAGCTAGCGGATCCCCGTTATCAGCAGA |  |
|  | Reverse | CATGCAGATCTTTCCTCGTGGACTGCACAG |  |
| **Mutation** |  | **Sequences (5’-3’)** |  |
| *Egfl7 mut1* | Sense | CAGAGAACACACACAttAAGTCCGGCGGGAAG |  |
|  | Antisense | CTTCCCGCCGGAC*TT*aaTGTGTGTGTTCTCTG |  |
| *Egfl7 mut2* | Sense | GCGGGAAGGGCCC*ATtt*CAGTCCTGATTTACC |  |
|  | Antisense | GGTAAATCAGGACTG*aa*ATGGGCCCTTCCCGC |  |
| *Flk1 p/e deletion mutant* | Sense | CCTTTTCCTCCATTTTTTCCTATTGGATCC |  |
|  | Antisense | GGATCCAATAGGAAAAAATGGAGGAAAAGG |  |
| *Flk1 p/e mut1* | Sense | AATCAGCAATTTAGgaAGATCTGTGCATCC |  |
|  | Antisense | GGATGCACAGATCTtcCTAAATTGCTGATT |  |
| *Flk1 p/e mut2* | Sense | GTCAGTGGGCCTGAGATtgTCAGATGGAGGTTCATC |  |
|  | Antisense | GATGAACCTCCATCTGAcaATCTCAGGCCCACTGAC |  |
| *Flk1 p/e mut3* | Sense | GGACTGGGGCAAAGgaAATCCCACCTTTAT |  |
|  | Antisense | ATAAAGGTGGGATTtcCTTTGCCCCAGTCC |  |

**Table S3. Primer sequences**
