# Supplementary material for: ETV2/ER71 regulates the generation of FLK1+ cells from mouse embryonic stem cells through miR-126-MAPK signaling
Source: Stem Cell Res Ther. 2019 Nov 19;10:328. doi: 10.1186/s13287-019-1466-8 (PMC6862833; doi:10.1186/s13287-019-1466-8)
Supplement: Supplementary file 3 — Additional file 3: Figure S1. Analysis on miR-126-5p in response to ETV2. Differentiated iFLAG-ETV2 mESCs at day 3.5 were subjected to qRT-PCR analysis. n=3, **p<0.01. [file 13287_2019_1466_MOESM3_ESM.docx]

**Figure S1. Analysis on miR-126-5p in response to ETV2.**  Differentiated iFLAG-ETV2 mESCs at day 3.5 were subjected to qRT-PCR analysis. n=3, ***p*<0.01.
